# Mother-Child Communication: The Influence of ADHD Symptomatology and Executive Functioning on Paralinguistic Style

**DOI:** 10.3389/fpsyg.2016.01203

**Published:** 2016-08-10

**Authors:** Elizabeth S. Nilsen, Ami Rints, Nicole Ethier, Sarah Moroz

**Affiliations:** ^1^Centre for Mental Health Research, Department of Psychology, University of Waterloo, WaterlooON, Canada; ^2^Department of Psychology, University of Western Ontario, LondonON, Canada

**Keywords:** paralinguistic, parent–child, executive functioning, inhibitory control, communication, pragmatic language, ADHD, dyadic analyses

## Abstract

Paralinguistic style, involving features of speech such as pitch and volume, is an important aspect of one’s communicative competence. However, little is known about the behavioral traits and cognitive skills that relate to these aspects of speech. This study examined the extent to which ADHD traits and executive functioning (EF) related to the paralinguistic styles of 8- to 12-year-old children and their mothers. Data was collected via parent report (ADHD traits), independent laboratory tasks of EF (working memory, inhibitory control, and cognitive flexibility), and an interactive problem-solving task (completed by mothers and children jointly) which was coded for paralinguistic speech elements (i.e., pitch level/variability; volume level/variability). Dyadic data analyses revealed that elevated ADHD traits in children were associated with a more exaggerated paralinguistic style (i.e., elevated and more variable pitch/volume) for both mothers and children. Mothers’ paralinguistic style was additionally predicted by an interaction of mothers’ and children’s ADHD traits, such that mothers with elevated ADHD traits showed exaggerated paralinguistic styles particularly when their children also had elevated ADHD traits. Highlighting a cognitive mechanism, children with weaker inhibitory control showed more exaggerated paralinguistic styles.

## Introduction

The phrase, ‘Use your inside voice!’ is frequently uttered within homes, and highlights our expectations for individuals to communicate in a particular manner. An individual’s style of speaking includes several paralinguistic features (e.g., pitch, tempo, and sound intensity; Cutler et al., 1997), and is implicated in several aspects of communicative competence (i.e., pragmatic language skills) including conveying sensitivity to situational context and/or listener characteristics (e.g., Ferguson, 1964; Kemper et al., 1995; Biersack et al., 2005) and providing important information about one’s intended meaning (Berman et al., 2010, 2013). Moreover, style of speech is a cue listeners rely on to form impressions about the personality characteristics of the speaker (Hecht and LaFrance, 1995). While a number of studies have sought to explicate children’s sensitivity to, or production of, particular speech styles (e.g., Morton and Trehub, 2001, Tomasello and Mannle, 1985; Weppelman et al., 2003), the degree to which individual differences in cognitive and behavioral traits relate to manner of speech has been neglected. As such, the present work assessed the degree to which child and maternal ADHD traits related to their paralinguistic style during an interactive problem-solving task – and whether children’s executive function skills accounted for any potential effects.

From an early age, children show sensitivity to the paralinguistic aspects of language. For example, 7-month-olds are able to discriminate sounds based on their frequency and harmonic structure (Clarkson and Clifton, 1985) and young infants prefer to listen to infant-directed speech that has increased prosodic qualities over adult-directed speech (Fernald, 1985; Cooper and Aslin, 1990). Mothers use different paralinguistic styles to convey different intentions, including to comfort and to show affection or approval (Fernald, 1993; Kitamura and Burnham, 2003), which infants can detect (Kitamura and Lam, 2009). Children’s interpretations of statements favor paralinguistic cues over lexical cues within the first year of life. Lexical cues are increasingly relied on during the mid-school years, and then, following this period, paralinguistic cues are privileged over lexical information once more (Friend, 2001; Morton and Trehub, 2001). With respect to the production of various paralinguistic cues, using a naturalistic observation, Tomasello and Mannle (1985) found that preschoolers used infant-directed intonation when interacting with their infant siblings, providing some evidence that preschoolers modify their paralinguistic behavior. However, Weppelman et al. (2003), using computer software to extract paralinguistic information, found that 4-year-olds did not modify their pitch or volume depending on their communicative partner (adult or child; Weppelman et al., 2003).

The ability to monitor and regulate one’s paralinguistic cues is viewed as a reflection of communicative competence (O’Neill, 2007). Moreover, research in the interpersonal domain suggests that paralinguistic behavior has been identified as a major contributor to how individuals form judgments and impressions about others. For instance, exposure to even 5 s of audio allows individuals to form personality (Hecht and LaFrance, 1995) and psychopathology ratings (Friedman et al., 2006; Fowler et al., 2009). These “thin slice” studies present audio clips that are too short to offer any kind of meaningful speech content; thus, one can infer that ratings are based primarily on paralinguistic cues. Given the importance of this aspect of language for children’s communicative development as well as for individuals’ interactions generally, it would be of interest to examine potential cognitive and behavioral factors that relate to particular speech styles.

Recently, there has been a growing emphasis on elucidating the cognitive mechanisms and behavioral traits that underlie communicative competence, broadly construed. For example, research has suggested that several aspects of communicative behavior are supported by underlying executive functioning (EF). EF refers to higher order processes that aid in the monitoring and control of thoughts/actions and facilitate goal-directed behavior (Burgess, 1997). Though different conceptualizations of EF exist (e.g., Jurado and Rosselli, 2007), core EF skills include inhibitory control (i.e., the ability to suppress dominant responses), working memory (i.e., actively holding important information in mind), and cognitive flexibility/shifting (i.e., considering simultaneous representations of an object or event and/or flexibly alternating between tasks), which are separable yet inter-related (e.g., Miyake et al., 2000; Huizinga et al., 2006). Individual differences in EF have been found to relate to the comprehension and production of communicative utterances in both pediatric (Nilsen and Graham, 2009, 2012; Gillis and Nilsen, 2014; Nilsen et al., 2015) and adult populations (Brown-Schmidt, 2009; Wardlow, 2013). Related to paralinguistic behavior more specifically, deficits in the *comprehension* of paralinguistic elements, such as prosody, have been associated with impairments in EF in various clinical samples, including those with depression (Uekermann et al., 2008) and traumatic brain injury (Struchen et al., 2008). However, the role of EF in the *production* of various paralinguistic features has not been clearly delineated and presents an important avenue for the present research.

The role of EF for communication poses important implications for individuals who show weaknesses in EF, such as amongst those with diagnoses of Attention-Deficit/Hyperactivity Disorder (ADHD; Barkley, 1997, 2006; Nigg, 2001; Martinussen et al., 2005; Willcutt et al., 2005), as well as those whose ADHD traits are at a sub-clinical threshold (Sonuga-Barke et al., 2002; Thorell and Wåhlstedt, 2006). That is, it may be that individuals with elevated ADHD traits show communicative styles that differ from those with low levels of ADHD traits due to differences in EF. Certainly, elevated levels of ADHD traits have also been associated with communicative difficulties across both parent-report (e.g., Bishop and Baird, 2001; Geurts et al., 2010) and performance-based communicative tasks (e.g., Purvis and Tannock, 1997; Nilsen et al., 2013). For example, children with elevated ADHD traits tend to provide insufficient information for listeners (Purvis and Tannock, 1997; Nilsen et al., 2015) and show difficulty with moderating the content of their utterances to fit the social context (Whalen et al., 1979; Landau and Milich, 1988). Critically, theoretical accounts have posited an underlying role of EF in accounting for these associations (e.g., Geurts et al., 2010; Nilsen and Fecica, 2011; Green et al., 2014). To date, however, little research has specifically examined the association of ADHD traits and paralinguistic aspects of speech. In addition, the available research has been limited to children’s detection of affect from paralinguistic cues (e.g., Uekermann et al., 2010; Oerlemans et al., 2014) or has examined this aspect as part of a broader constellation of other communicative abilities (e.g., Kim and Kaiser, 2000). Resultantly, little is presently known about the specific qualities of speech that may differ for individuals with elevated ADHD traits, and more specifically, the extent to which EF may be implicated in any association between these factors.

Attempting to fill gaps in the literature, this study focused specifically on examining the paralinguistic styles of mothers and children during an interactive task. We isolated paralinguistic qualities, including the features of volume mean (the general volume at which one is speaking); volume variation (which can be thought of as vocal emphasis where greater volume variation suggests that a person is using punctuated bursts of volume in order to emphasize certain statements or words); pitch mean (the average fundamental frequency at which one speaks); and pitch variation (the degree to which an individual’s pitch rises and falls over time). We chose to assess these speech qualities during a mother–child interaction as previous work has highlighted the degree to which ADHD traits, in either a mother or a child, impact content of the interpersonal exchange. For example, mothers with increased ADHD symptoms demonstrate less corrective feedback to their children and use shorter, less complex, and less elaborate language with their children (Kryski et al., 2010; Griggs and Mikami, 2011), while children with increased ADHD symptoms tend to have parents who use more negative-reactive and less positive parenting strategies, and are more directive, controlling, and negative (Mash and Johnston, 1982; Johnston, 1996; Gadeyne et al., 2004). In the present study, our first research aim was to examine whether children’s and mothers’ ADHD traits related to their paralinguistic style. In particular, we wondered whether elevated ADHD traits would result in a more exaggerated style (i.e., higher and more variable pitch and volume) given that there may be less regulation of vocal behavior. Dyadic data analyses (i.e., Actor-Partner Interdependence Model; APIM) were used to examine and account for the extent to which one’s ADHD traits would relate to one’s own paralinguistic style, as well as the paralinguistic style of one’s partner (Kenny et al., 2006). Such an approach is important as it allowed for us to account for the interdependence in the relationship between mothers and their children when assessing the relations between ADHD and paralinguistic style (Cook and Kenny, 2005). We also examined the extent to which child and maternal ADHD traits may interact to impact either or both partners’ paralinguistic style (as per previous work suggesting that social interactions differ depending on the degree of similarity/difference between parent and child symptomatology; e.g., Psychogiou et al., 2007, 2008; Wymbs et al., 2015). That is, we questioned whether having both a parent and child with elevated ADHD traits could lead to a differential paralinguistic style (relative to when only one member had elevated traits).

The second research aim was to determine whether, for children, EF accounted for a more exaggerated paralinguistic style. Accordingly, regression analyses were conducted to assess the degree to which children’s performance on EF tasks (assessing working memory, inhibitory control, and cognitive flexibility) related to a paralinguistic style that was more exaggerated (i.e., using ADHD traits and EF as predictors of the paralinguistic composite). Note that while we were interested in assessing whether children’s EF skills may be responsible for the relation between ADHD, mediation analyses were not conducted due to the assumption of causality that is implicit within mediation analyses. That is, mediation analysis implies that the mediator is ‘caused’ by the predictor (Baron and Kenny, 1986). Recent theoretical accounts posit that executive deficits may give rise to ADHD symptoms, rather than being a consequence of the disorder (Crosbie et al., 2008). In addition, other work shows that, while relations do exist, there is much variability in the degree of EF impairment for youth with ADHD (Lambek et al., 2011). Thus, we looked at these factors concurrently rather than assuming one was a downstream effect of another.

## Materials and Methods

### Participants

Participants consisted of 68 pairs of community-recruited mothers and children (aged 8–12 years). Forty-three children were reported to be typically developing, while 25 were reported to have received a previous diagnosis of ADHD from a physician (2), pediatrician (9), or psychologist (16; participants were allowed to select more than one option). As ADHD traits operate on a continuum and exist within non-clinical samples (e.g., Bignell and Cain, 2007), children with and without a previous diagnosis of ADHD were able to participate. Participants were recruited from a community reflecting typically middle class families using flyers and advertisements posted at local community centers and internet sites promoting child-focused programs. The majority of participants were Caucasian (85%) with all participants reporting fluency in English and English reported as the primary language for 96% of participants (other languages included Mandarin, Urdu, and Taiwanese). Nine dyads were excluded from analyses due to a microphone malfunction and two dyads due to statistical outliers (i.e., >3*SD*; one on the cognitive flexibility task and another on the child’s paralinguistic data). This resulted in a final sample of 57 dyads (24 females, *M*age = 10 years; 4 months [*SD* = 14.58 months]; see **Table 1**). Twenty-two of the remaining children had previous diagnoses, and 20 of these children were prescribed stimulant medication for ADHD traits (with 11 taking their medication on the day of testing^1^).

**Table 1:** Demographic information and executive function task performance.

	*M* (*SD*)
Age	10 years; 4 months (14.58 months)
Gender	Males: 57.9% (*n* = 33); Females: 42.1% (*n* = 24)
Percentage of children with a previous diagnosis of ADHD	38.6% (*n* = 22)
Percentage of previously diagnosed children on medication at time of testing	50% (*n* = 11)
Children’s SNAP scores	Inattention: 1.15 (0.86; range: 0–3)
	Hyperactivity: 0.76 (0.77; range: 0–2.89)
Mothers’ CAARS scores (*T*-scores)	Inattention: 52.38 (12.54; range: 36.00–88.50)
	Hyperactivity: 50.07 (9.64; range: 35.50–73.50)
Correct responses on Stroop Task	77.28 (17.72; range: 41–110)
Correct responses on Reading Span Task	19.11 (2.04; range: 14–25)
Response Time on Trails Letter–Number	127.96 s (68.96 s; range: 46–358)
Errors on Trails Letter–Number	1.12 (1.46; range: 0–6)


### Materials and Procedures

The present research was part of a larger study on children’s communicative behavior and consisted of one 90-min session for each dyad. This study was approved through the Office of Research Ethics at the University of Waterloo. Mothers completed questionnaires independently while children completed the EF tasks with a researcher in a separate room. All tasks were presented in a fixed order, a standard practice when assessing individual differences that ensures that individuals are exposed to identical stimulus contexts. Subsequently, the mothers and children participated in an interactive task together.

#### ADHD Traits

Children’s ADHD traits were assessed via parent-report using the Inattention and Hyperactivity-Impulsivity subscales of the SNAP-IV Teacher and Parent Rating Scale (SNAP-IV; Swanson, 1992). Mothers rated the extent to which children demonstrated 18 behaviors from (0) *Not At All* to (3) *Very Much.* The SNAP-IV has acceptable internal consistency, item selection, and factor structure, all consistent with the constructs of ADHD put forth in the *Diagnostic and Statistical Manual of Mental Disorders, Fourth Edition* (*DSM-IV*; Bussing et al., 2008). Mothers’ ADHD traits were assessed through mothers’ self-report on the Conners Adult ADHD Rating Scale – Short version (CAARS-S; Conners et al., 1999). Mothers rated the frequency with which they exhibited 26 behaviors related to ADHD on a scale from (0) *Not At All* to (3) *Very Much/Very Frequently*. The Conners scale demonstrates good internal consistency and predictive validity (Erhardt et al., 1999; Kooij et al., 2013).

In the present study, strong positive correlations emerged between the inattentive and hyperactive subscales of the SNAP-IV (*r* = 0.79, *p* < 0.001) and the CAARS-S (*r* = 0.47, *p* = 0.02). Given this, and consistent with current factor analytic studies supporting the use of a general factor of ADHD (e.g., Normand et al., 2012), scores across inattentive and hyperactive-impulsive traits were averaged to create a single estimate of ADHD traits for children and mothers. For all analyses, ADHD traits were treated continuously, consistent with research suggesting that ADHD traits fall on a continuum in community samples (Bignell and Cain, 2007).

#### Executive Function

Developmentally appropriate tasks to capture each aspect of EF (inhibitory control, working memory, cognitive flexibility were selected.

##### Inhibitory control

Children’s inhibitory control, specifically the interference control aspects of inhibitory control, was assessed using a Stroop task (Stroop, 1935). This task asked children to first read words from a list of colors with congruent font colors and then identify the colors of the font from a list of words with incongruent font colors. Stroop tasks have been found to load onto an inhibition factor in factor analytic studies (Miyake et al., 2000). The task discontinued after 2 min for all children. Interference control was calculated by regressing the incongruent color-word naming scores on the congruent color naming scores and saving the unstandardized residuals wherein higher scores reflect better performance.

##### Working memory

Children’s working memory was assessed using the reading span task from the Stanford–Binet intelligence scales (Roid, 2003). This task has demonstrated acceptable reliability and strong correlations with other measures of working memory (Conway et al., 2005). Children were asked to answer sets of yes/no questions (asked by the examiner) and recall the last word in each question (with the number of questions in each set increasing over trials). Scores reflect the number of errors made, ranging from 0 (two or more errors) to 2 (no errors). Per task instructions, testing discontinued when two consecutive scores of 0 were obtained. Participants’ total score was included in the analyses.

##### Cognitive flexibility

The Trail Making Test (Reitan and Wolfson, 1985) was used to examine children’s cognitive flexibility (Sánchez-Cubillo et al., 2009). Performance on this task has been shown to correlate with other measures of set-switching (Arbuthnott and Frank, 2000). Children began by connecting circles of sequential numbers on paper; they then switched to connecting alternated numbers and letters, again in sequential order, with this latter task reflecting children’s ability to flexibly switch sequences. Ratio scores were calculated to index children’s time to complete the second task controlling for time to complete the first task, and were entered into the analyses (Strauss et al., 2006).

#### Mother–Child Interaction

After the questionnaires and EF tasks were completed, the mother joined her child in a second testing room where they sat side-by-side (positioning in terms of left/right was randomly chosen by dyads). Dyads were provided with a relatively open-ended collaborative problem-solving task based on a task used by youth groups (Scouting Web, 2013), and which required dyads to imagine they were survivors of an airplane crash in freezing temperatures and 20 miles from the nearest town. They were informed that in the plane wreckage they would find 10 items (shown by pictures, i.e., a compass, gun, cooking oil, lighter, heavy-duty canvas, map, ax, steel wool, chocolate bars, and newspaper), and were asked to work together to generate one list rating the items from most to least important. Pairs were given 15 min to complete the task, and were asked to converse in their typical fashion if they finished early. The interaction was recorded by way of a microphone, placed in a designated position on the table between the dyad, connected wirelessly to a video camera located behind a one-way mirror.

##### Extraction of auditory data

To obtain measures of paralinguistic styles for children and their mothers, each video was converted into an auditory file using the VLC media player (VideoLAN, 2012). Researchers then selected samples of interactants’ speech from a 10-min window of the interaction during which mothers and children worked on the task. The researcher compiled a selection of 10 speech clips using the program Audacity (Audacity Team, 2011), using a standardized sampling procedure: one clip from each of the first 10 min of the interaction was extracted, beginning from 30 s after the experimenter left the room (i.e., 30 s, 1 min 30 s, 2 min 30 s, etc.). To be viable, an audio clip had to involve the participant speaking and be least 2 s long, to a maximum of 10 s. An audio clip was deemed unusable when it involved a participant reading from the information sheet (i.e., not producing spontaneous speech), when it was interrupted by the partner’s voice, and when it contained audible background noise. The voice clips were then compiled to form a 30–60 s sample of the participant’s paralinguistic style. If the resulting compilation did not exceed 30 s, the researcher sampled the 11th and 12th minutes of the interaction, under the condition that the mother and child were still working on the task. Failing this, the researcher re-sampled the first 10 min (i.e., using a second clip within the minute time frames), until at least 30 s of material could be obtained. These paralinguistic samples were compiled for both the mother and the child, separately by two different researchers. The computer program Praat (Boersma and Weenink, 2011) was then used to determine the mean and standard deviation of both pitch (in Hertz) and volume (in decibels) of the paralinguistic sample. The various indices (i.e., pitch and volume mean and standard deviation) were standardized due to differences in the scaling and then aggregated to provide an index of exaggerated paralinguistic styles (i.e., wherein higher scores were reflective of a communicative style evidenced by elevated and more variable pitch and volume). Intercorrelations among children and mothers’ paralinguistic qualities, as well as their relation to the overall composite are provided on **Table 2.** Cronbach’s alpha for the exaggerated paralinguistic composite was 0.69 for children and 0.60 for mothers.

**Table 2:** Intercorrelations amongst paralinguistic qualities in children’s and mothers’ speech.

	Pitch Mean	Pitch *SD*	Volume Mean	Volume *SD*
Children				
Pitch Mean	–			
Pitch *SD*	0.14	–		
Volume Mean	0.45^∗∗^	0.15	–	
Volume *SD*	0.54^∗∗^	0.30^∗^	0.44^∗∗^	–
Exaggerated Composite	0.79^∗∗^	0.42^∗∗^	0.76^∗∗^	0.83^∗∗^
Mothers				
Pitch Mean	–			
Pitch *SD*	0.30^∗^	–		
Volume Mean	0.40^∗∗^	0.36^∗∗^	–	
Volume *SD*	0.24^†^	0.13	0.24^†^	–
Exaggerated Composite	0.69^∗∗^	0.62^∗∗^	0.76^∗∗^	0.63^∗∗^


*^†^*p* < 0.10, ^∗^*p* < 0.05, ^∗∗^*p* < 0.01 (all two-tailed).*

## Results

### Preliminary Analyses

**Table 1** provides the demographic characteristics of the sample. The ratings of mothers’ and children’s ADHD traits showed a wide range that was normally distributed and within acceptable limits for skew/kurtosis. Mean levels of ADHD were within the non-clinical range. There were no significant correlations between children’s age and ADHD traits (*p* = 0.83), but age was correlated with exaggerated paralinguistic style, *r* = -0.30, *p* = 0.03, and as such was controlled in further analyses. A *t*-test revealed no significant difference between male and female children on the exaggerated paralinguistic aggregate, *p* = 0.82, nor was gender significantly associated with any other key variables included in the study, *p*s > 0.09. As such, gender was not included in further analyses.

The bivariate correlation between the mothers’ and children’s paralinguistic behavior was significant, *r* = 0.40, *p* = 0.003 (*r* = 0.43, *p* = 0.001 when controlling for child’s age), suggesting that a dyadic approach to the analyses is important given the influence that mother/child paralinguistic behaviors have on each other.

### Relation between Paralinguistic Style and ADHD Traits

**FIGURE 1:**
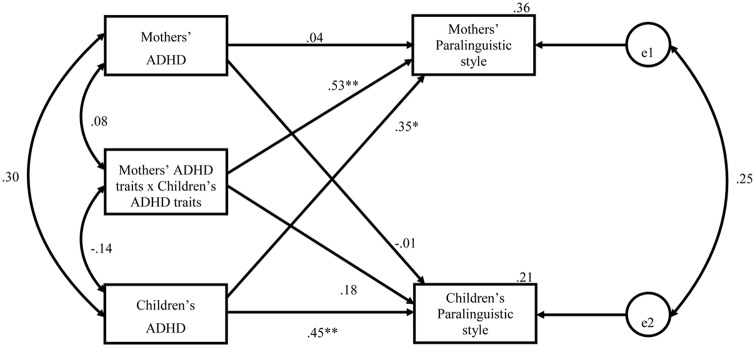
**APIM: ADHD traits predicting exaggerated paralinguistic style (standardized estimates)**.

Actor-Partner Interdependence Model analyses (using AMOS Graphics 22.0; Arbuckle, 2013) were used to examine the association between paralinguistic style and ADHD traits. Such analyses allow for investigating both actor effects (i.e., how one’s ADHD traits predict their own paralinguistic style, shown in **Figure 1** as horizontal lines representing the standardized regression weights), and partner effects (i.e., how one’s ADHD traits predict one’s partner’s paralinguistic style, shown as diagonal lines in **Figure 1**) (Kenny et al., 2006), as well as to assess the effects of an interaction between mothers’ and children’s ADHD traits on each partner’s respective paralinguistic style. Moreover, this model allowed us to examine the correlation between maternal and child ADHD traits (i.e., the arc on the left of the model represents the covariation between mothers’ and children’s ADHD traits) and the correlation between maternal and child paralinguistic styles (i.e., the arc on the right of the model represents the covariation between the residual variance of mothers’ and children’s paralinguistic styles) controlling for the predictors. All predictor variables were mean centered prior to analyses.

Addressing our first research question, the analyses revealed a significant actor effect for children, such that increased ADHD traits were significantly associated with a more exaggerated paralinguistic style, β = 0.45, *p* = 0.001. In addition, a significant partner effect emerged for mothers, such that increased ADHD traits in children were significantly associated with a more exaggerated paralinguistic style in mothers, β = 0.35, *p* = 0.012. Thus, increased child ADHD traits related to more exaggerated paralinguistic styles in both children and mothers.

**FIGURE 2:**
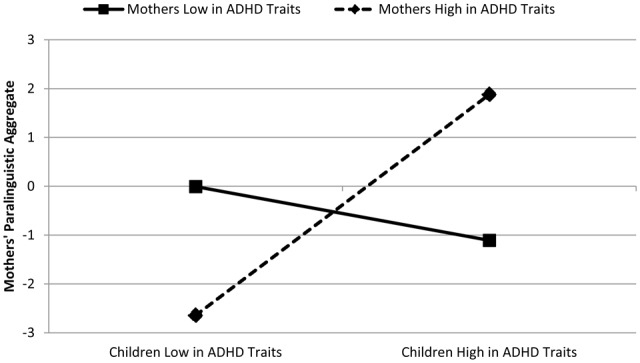
**Mothers’ and children’s ADHD traits predicting mothers’ exaggerated paralinguistic style**.

The pattern for maternal ADHD traits showed a more complex pattern. That is, we did not find that the ADHD traits of mothers related to the paralinguistic style of their children (β = -0.01, *p* = 0.97), nor was the interaction term a significant predictor of children’s paralinguistic aggregates (β = 0.18, *p* = 0.28). However, a significant interaction effect emerged with respect to mother’s paralinguistic style, β = 0.53, *p* < 0.001 (**Figure 2**). Tests of the simple slopes suggested that when mothers’ ADHD traits were low, children’s ADHD traits did not influence mothers’ paralinguistic style (β = -0.18, *p* = 0.42). However, when mothers’ ADHD traits were high, having a child who demonstrated low levels of ADHD traits was associated with a more subdued paralinguistic style in mothers, while having children with higher levels of ADHD traits was associated with a more exaggerated paralinguistic style in mothers (β = 0.86, *p* = 0.002).

The percentage of variance in the paralinguistic aggregate explained by ADHD traits (i.e., including children’s ADHD traits, maternal ADHD traits, and the interaction of the two) was 21% for children and 36% for mothers. In terms of other findings, we did not find a significant correlation between ADHD traits in mothers and children (*r* = 0.30, *p* = 0.14), suggesting that mothers did not tend to report comparable levels of ADHD traits for themselves and their children. In addition, once controlling for ADHD traits, the correlation between mothers’ and children’s paralinguistic aggregates was not significant (*r* = 0.25, *p* = 0.14).

### Children’s Executive Functioning and Paralinguistic Style

Correlations between children’s ADHD traits, EF, and the paralinguistic aggregate are shown in **Table 3.** Children’s ADHD traits were related to their inhibitory control skills *(r* = -0.37, *p* = 0.005), but not working memory or cognitive flexibility (*p*s > 0.54).

**Table 3:** Bivariate correlations between children’s ADHD traits, executive functioning, and paralinguistic style (partial correlations controlling for age in months are in parentheses).

	ADHD traits	Inhibitory control	Working memory	Cognitive flexibility	Exaggerated paralinguistic Style
Age (months)	-0.03	0.44^∗∗^	0.21	0.10	-0.30^∗^
ADHD traits		-0.37^∗∗^ (-0.40)^∗∗^	-0.08 (-0.06)	-0.08 (-0.09)	0.42^∗∗^ (0.43^∗∗^)
Inhibitory control			0.36^∗∗^ (0.32^∗^)	0.03 (0.01)	-0.51^∗∗^ (-0.44^∗∗^)
Working memory				-0.14 (-0.12)	-0.11 (-0.06)
Cognitive flexibility					0.01 (-0.03)


*^∗^*p* < 0.05, ^∗∗^*p* < 0.01 (all two-tailed).*

Addressing the second research question, hierarchical regression analyses were conducted to examine the extent to which EF was related to paralinguistic style. At the first step of the regression, ADHD traits were a significant predictor of paralinguistic style (β = 0.41, *p* = 0.001), as was age (β = -0.29, *p* = 0.02). The regression model with these two predictors accounted for a significant amount of variance in children’s paralinguistic style, *R*^2^ = 0.26. When inhibitory control was entered alongside age and ADHD traits in the second step, there was a significant increase in the variance explained by the model, where the change in *R*^2^ = 0.08 (together accounting for 34% of the variance). Both ADHD traits and inhibitory control were significant predictors of children’s paralinguistic style (inhibitory control, β = -0.34, *p* = 0.01; ADHD traits, β = 0.29, *p* = 0.02). Thus, children with elevated ADHD traits, as well as those with weaker inhibitory control, show a more exaggerated paralinguistic style, with both characteristics accounting for unique variance. (Of note, Fischer’s *z*-tests were conducted to examine whether there were significant differences between ADHD traits and each specific paralinguistic feature (controlling for age). Volume variability was found to have a stronger relation with ADHD traits than all other paralinguistic features (*p*s < 0.02), which did not differ from each other (*p*s > 0.36). In addition, Fisher’s *z*-tests revealed no significant differences in the strength of the relations between inhibitory control and each paralinguistic feature, controlling for age (*p*s > 0.19)).

Similar regression analyses were conducted that included both working memory and cognitive flexibility in the place of inhibitory control, yielding non-significant results. That is, working memory and cognitive flexibility did not significantly improve the model containing just ADHD traits and age (consistent with the non-significant bivariate correlations between these measures and paralinguistic style).

## Discussion

Given the relevance of paralinguistic style for both communicative development and impression formation, it is important to understand individual differences that may contribute to variability in speech styles. The present study provides insight into the degree to which maternal and child ADHD traits, and children’s EF skills, related to their paralinguistic style.

First, we found that elevated ADHD traits in children related to the production of a more exaggerated paralinguistic style; that is, speaking in a louder, higher-pitched, and more variable fashion (which was particularly characterized by greater volume variability). This was true even after controlling for mothers’ paralinguistic style (i.e., to account for possible phonetic-acoustic convergence effects; Pardo, 2013). These findings suggest that in addition to affecting the *content* of children’s utterances (e.g., Purvis and Tannock, 1997; Nilsen et al., 2015), children’s ADHD traits also relate to their manner of speaking. Past work has found that elevated ADHD traits result in worse detection of affective prosody recognition (albeit in a sample of children with Autism Spectrum Disorder; Oerlemans et al., 2014). As such, it may be that the children in our study with elevated ADHD traits are less sensitive to their own paralinguistic style, thereby having more difficulty with regulating their communicative behavior. While previous work has found that children with elevated traits show differential communicative behavior, this research has either not isolated paralinguistic style from other communicative behavior (Kim and Kaiser, 2000) or has relied on global parent report measures of communication (Leonard et al., 2011), which limits conclusions about how ADHD traits impact paralinguistic styles specifically (though certainly, examining the relation between paralinguistic style and other communicative behavior would be an interesting extension of this work). Moreover, in the present work, we used an objective computerized coding system to isolate and capture the paralinguistic style. By doing so, we could ensure that the measures were not impacted by the overt behavior of the child (which poses problems for interpreting communicative skills based on observer data).

In addition to an actor effect, children’s ADHD traits were associated with a more exaggerated paralinguistic style in mothers, which adds to our understanding of features which characterize communication between children with traits of ADHD and others in their social worlds. That is, even when the mother’s own style and behavioral profile were controlled, we found that the behavioral traits of the child related to a maternal paralinguistic style that was more exaggerated. The impact of child symptomatology on a parents’ behavior has been explored previously, showing that elevated ADHD traits negatively impact parents’ parenting styles (e.g., Mash and Johnston, 1982; Johnston, 1996; Gadeyne et al., 2004). What is unique about the present findings is that we find that child ADHD traits influence a very basic, yet important, component of a mother’s interaction style. A more stringent extension of these findings would be to determine whether mothers vary their paralinguistic style when interacting a child who has ADHD versus another child who does not.

Second, we found an interaction effect, such that mothers’ ADHD traits had a differential relation to their own paralinguistic style depending on the level of ADHD traits exhibited by their children. More specifically, it seems that when mothers’ ADHD traits were low, children’s ADHD traits did not seem to impact mothers’ paralinguistic style. In contrast, when maternal ADHD traits were high, there was an impact of children’s ADHD traits on maternal paralinguistic style. More specifically, for mothers with high ADHD traits, when children’s ADHD traits are low, the paralinguistic style was more subdued, whereas when children’s ADHD traits are high, mothers’ paralinguistic style was more exaggerated (i.e., increased and more variable prosody and volume). Thus, it seems as though mothers with elevated ADHD traits are more impacted by the behavioral styles of their children, which are manifest through their paralinguistic styles. These findings are interesting to consider in the context of past work examining the “similarity-fit hypothesis” which posits that parents and children may have better interactions when there is a similarity in ADHD symptomatology. Conflicting support for such hypotheses have been yielded, whereby some studies, supporting the similarity-fit predictions, have found that high maternal traits ameliorate the negative effects of child ADHD on parenting (e.g., Psychogiou et al., 2007, 2008). In contrast, others have found evidence in support of “similarity-misfit” predictions such that parent and child ADHD symptoms operate together to predict negative parenting styles and communicative patterns (Wymbs et al., 2015). In the present work, we find that there is indeed an interaction between maternal and child ADHD traits for mothers’ paralinguistic style. That is, mothers with elevated ADHD traits, when interacting with a child with elevated ADHD traits, are showing a similar exaggerated paralinguistic style. However, the current data do not allow for determining whether reciprocity in the exaggerated paralinguistic style is beneficial (e.g., sharing a similar exuberant style) or maladaptive (e.g., both engaging in a less regulated style). That is, while reciprocity in other aspects of interaction (e.g., affiliation) is seen as resulting in more satisfying and positive interactions (e.g., Tracey, 2004; Locke and Sadler, 2007), it is difficult to know how similarly matched exaggerated paralinguistic styles would be experienced by the interlocutors.

Third, we found that inhibitory control contributed to children’s manner of speaking. That is, children with weaker inhibitory control (but not working memory or cognitive flexibility) showed a more exaggerated paralinguistic style. Such a finding suggests that an individual’s paralinguistic style is something that is, at least in part, reliant on cognitive resources. Moreover, such a finding allows for speculation about the meaning of the exaggerated style – that is, that it may reflect a less controlled, more chaotic manner of speaker as opposed to a controlled, but more exuberant or excited style. However, as noted above, the impact for such a paralinguistic style for impression formation or interpersonal interactions cannot be determined from our data and will be an important avenue for future research. Regardless, findings add to a growing body of work highlighting the role of inhibitory control for other aspects of communication, such as suppressing one’s own perspective in order to take the perspective of a conversational partner (Nilsen and Graham, 2009, 2012). Interestingly, we found that although ADHD traits and inhibitory control were related (as has been demonstrated previously, Sonuga-Barke et al., 2002; Thorell and Wåhlstedt, 2006), each characteristic accounted for unique variance in children’s paralinguistic style. Thus, it could be the case that children who behaviorally exhibit ADHD traits and/or cognitively show difficulties with suppressing dominant reactions would show a paralinguistic style that is more exaggerated than other children.

The present work advances the understanding of the role of ADHD traits and inhibitory control in communicative behavior. However, a number of limitations should be noted. First, the functional impact of an exaggerated paralinguistic style, and more specifically how it may impact one’s social functioning, cannot be ascertained from this study. That is, it would be of interest for future work to examine how others interpret an exaggerated paralinguistic style and whether such a style impacts the reaction of one’s communicative partner. Certainly, there is some emerging work suggesting that paralinguistic styles influence adults’ ratings of speakers’ socio-communicative competency (Varghese and Nilsen, 2015). Moreover, an important extension of this work would be to determine whether ADHD traits or inhibitory control relate to the extent to which individuals are able to appropriately modify their paralinguistic styles across contexts (Ellis and Ure, 1969). Second, we did ask about nor conduct an examination of the hearing abilities of our sample. Third, our findings are limited to mothers and mother–child interactions and may not generalize to father–child dyads given significant variability in vocal qualities across genders (e.g., Warren-Leubecker and Bohannon, 1984). Fourth, EF skills of mothers, as well as their age, were not examined, and thus the role of inhibitory control in the paralinguistic style of adults and the possible influence of maternal age has been left for future work to explore. Finally, to minimize the length of the study we had only included one task of EF for each component; however, such a decision prevented us from analyzing the data using latent variables for the various EF constructs.

In sum, the paralinguistic features of one’s speech represent an important aspect of one’s communicative approach. The present work highlights the role of ADHD traits and inhibitory control for a paralinguistic style that is more exaggerated and unrestrained. While previous studies have found that these individual factors relate to the *content* of communication, this is the first to show their importance in predicting an individual’s manner of speech. Findings have implications for theoretical models of communication, which identify executive functions as an important factor in the development of children’s communicative skills, as well adding to the growing literature as to how ADHD traits relate to one’s own as well as others’ communicative behavior.

## Author Contributions

EN participated in/supervised the design, data collection, analyses, and manuscript-writing; AR analyzed the data and contributed to the conceptual framework in writing the manuscript; NE designed the study, collected data, and contributed to the writing of the manuscript; SM collected data as part of her honors thesis and contributed to the writing of the manuscript.

## Conflict of Interest Statement

The authors declare that the research was conducted in the absence of any commercial or financial relationships that could be construed as a potential conflict of interest.

## Footnotes

1 It was requested that children not take their medication on the day of testing, but it was made clear the decision was up to the parent/child and would not affect their ability to participate in the study. Medication status was not significantly related to the dependent measures (i.e., when comparing the performance of those individuals with a previous diagnosis of ADHD who were taking versus not taking medication; *p* = 0.25) and, as such, was not included in further analyses.

## References

[B1] Arbuckle J. L. (2013). *Amos (Version 22.0) [computer program].* Chicago: SPSS.

[B2] Arbuthnott K. Frank J. (2000). Trail making test, part B as a measure of executive control: validation using a set-switching paradigm. *Clinical and Experimental Neuropsychology* 22 518–528. 10.1207/S15324826AN0902_5 10923061

[B3] Audacity Team (2011). *Audacity (Version 1.3 beta) [computer software].* Available from http://audacity.sourceforge.net/

[B4] Barkley R. A. (1997). Behavioral inhibition, sustained attention, and executive functions: Constructing a unifying theory of ADHD. *Psychol. Bull.* 121 65–94. 10.1037/0033-2909.121.1.65 9000892

[B5] Barkley R. A. (2006). *Attention-Deficit Hyperactivity Disorder: A Handbook for Diagnosis and Treatment*, 3rd Edn New York, NY: Guilford Press.

[B6] Baron R. M. Kenny D. A. (1986). The moderator-mediator variable distinction in social psychological research: conceptual, strategic, and statistical considerations. *J. Pers. Soc. Psychol.* 51 1173–1182. 10.1037/0022-3514.51.6.1173 3806354

[B7] Berman M. J. Chambers C. G. Graham S. A. (2010). Preschoolers’ appreciation of speaker vocal affect as a cue to referential intent. *J. Exp. Child Psychol.* 107 87–99. 10.1016/j.jecp.2010.04.012 20553796

[B8] Berman M. J. Graham S. A. Chambers C. G. (2013). Contextual influences on children’s use of vocal affect cues during referential interpretation. *Q. J. Exp. Psychol.* 66 705–726. 10.1080/17470218.2012.713367 22950850

[B9] Biersack S. Kempe V. Knapton L. (2005). “Fine-tuning speech registers: a comparison of the prosodic features of child-directed and foreigner-directed speech,” in *Proceedings of the 9th European Conference on Speech Communication and Technology: Interspeech*, Lisbon, 2401–2404.

[B10] Bignell S. Cain K. (2007). Pragmatic aspects of communication and language comprehension in groups of children differentiated by teacher ratings of inattention and hyperactivity. *Br. J. Dev. Psychol.* 25 499–512. 10.1348/026151006X171343

[B11] Bishop D. V. M. Baird G. (2001). Parent and teacher report of pragmatic aspects of communication: use of the children’s communication checklist in a clinical setting. *Dev. Med. Child Neurol.* 43 809–818. 10.1017/S0012162205000435 11769267

[B12] Boersma P. Weenink D. (2011). *Praat (Version 5.3) [Computer Software].* Available from http://www.fon.hum.uva.nl/praat/

[B13] Brown-Schmidt S. (2009). The role of executive function in perspective taking during online language comprehension. *Psychon. Bull. Rev.* 16 893–900. 10.3758/PBR.16.5.893 19815795

[B14] Burgess P. W. (1997). “Theory and methodology in executive function and research,” in *Methodology of Frontal and Executive Function*, ed. Rabbitt P. (Hove: Psychology Press), 81–116.

[B15] Bussing R. Fernandez M. Harwood M. Hou W. Wilson Garvan C. Eyberg S. M. (2008). Parent and teacher SNAP-IV ratings of attention deficit hyperactivity disorder traits. *Assessment* 15 317–328. 10.1177/1073191107313888 18310593 PMC3623293

[B16] Clarkson M. G. Clifton R. K. (1985). Infant pitch perception: evidence for responding to pitch categories and the missing fundamental. *J. Acoust. Soc. Am.* 77 1521–1528. 10.1121/1.391994 3989107

[B17] Conners C. K. Erhardt D. Sparrow E. P. (1999). *Conners’ Adult ADHD Rating Scales: Technical Manual.* New York, NY: Multi-Health Systems.

[B18] Conway A. R. A. Kane M. J. Bunting M. F. Hambrick D. Z. Wilhelm O. Engle R. W. (2005). Working memory span tasks: a methodological review and user’s guide. *Psychon. Bull. Rev.* 12 769–786. 10.3758/BF03196772 16523997

[B19] Cook W. L. Kenny D. A. (2005). The actor-partner interdependence model: a model of bidirectional effects in developmental studies. *Int. J. Behav. Dev.* 29 101–109. 10.1080/01650250444000405

[B20] Cooper R. P. Aslin R. N. (1990). Preference for infant-directed speech in the first month after birth. *Child Dev.* 61 1584–1595. 10.2307/1130766 2245748

[B21] Crosbie J. Pérusse D. Barr C. L. Schachar R. J. (2008). Validating psychiatric endophenotypes: inhibitory control and attention deficit hyperactivity disorder. *Neurosci. Biobehav. Rev.* 32 40–55. 10.1016/j.neubiorev.2007.05.002 17976721

[B22] Cutler A. Dahan D. van Donselaar W. (1997). Prosody in the comprehension of spoken language: a literature review. *Lang. Speech* 40 141–201. 9509577 10.1177/002383099704000203

[B23] Ellis J. Ure J. (1969). “Language varieties: Register,” in *Encyclopedia of Linguistics: Information and Control*, ed. Meetham A. R. (London: Pergamon), 251–259.

[B24] Erhardt D. Epstein J. N. Conners C. K. Parker J. D. A. Sitarenios G. (1999). Self-ratings of ADHD traits in auts II: reliability, validity, and diagnostic sensitivity. *J. Atten. Disord.* 3 153–158. 10.1177/108705479900300304

[B25] Ferguson C. A. (1964). Baby talk in six languages. *Am. Anthropol.* 66 103–114. 10.1525/aa.1964.66.suppl_3.02a00060

[B26] Fernald A. (1985). Intonation and communicative intent in mothers’ speech to infants: is the melody the message? *Child Dev.* 60 1497–1510. 10.2307/1130938 2612255

[B27] Fernald A. (1993). Approval and disapproval: infant responsiveness to vocal affect in familiar and unfamiliar languages. *Child Dev.* 64 657–674. 10.2307/1131209 8339687

[B28] Fowler K. A. Lilienfeld S. O. Patrick C. J. (2009). Detecting psychopathology from thin slices of behavior. *Psychol. Assess.* 21 68–78. 10.1037/a0014938 19290767

[B29] Friedman J. N. W. Oltmanns T. F. Gleason M. E. J. Turkheimer E. (2006). Mixed impressions: reactions of strangers to people with pathological personality traits. *J. Res. Pers.* 40 395–410. 10.1016/j.jrp.2005.01.005

[B30] Friend M. (2001). The transition from affective to linguistic meaning. *First Lang.* 21 219–243. 10.1177/014272370102106302 PMC5509358 28713182

[B31] Gadeyne E. Ghesquière P. Onghena P. (2004). Longitudinal relations between parenting and child adjustment in young children. *J. Clin. Child Adolesc. Psychol.* 33 347–358. 10.1207/s15374424jccp3302_16 15136199

[B32] Geurts H. M. Broeders M. Nieuwland M. S. (2010). Thinking outside the executive functions box: theory of mind and pragmatic abilities in attention deficit/hyperactivity disorder. *Eur. J. Dev. Psychol.* 7 135–151. 10.1080/17405620902906965

[B33] Gillis R. Nilsen E. S. (2014). The role of cognitive flexibility in children’s ability to detect communicative ambiguity. *First Lang.* 34 58–71. 10.1177/0142723714521839

[B34] Green B. C. Johnson K. A. Bretherton L. (2014). Pragmatic language difficulties in children with hyperactivity and attention problems: an integrated review. *Int. J. Lang. Commun. Disord.* 49 15–29. 10.1111/1460-6984.12056 24372883

[B35] Griggs M. S. Mikami A. Y. (2011). The role of maternal and child ADHD traits in shaping interpersonal relationships. *J. Abnorm. Child Psychol.* 39 437–449. 10.1007/s10802-010-9464-4 20931275

[B36] Hecht M. A. LaFrance M. (1995). How (fast) can I help you? Tone of voice and telephone operator efficiency in interactions. *J. Appl. Psychol.* 25 2086–2098. 10.1111/j.1559-1816.1995.tb02389.x

[B37] Huizinga M. Dolan C. V. van der Molen M. W. (2006). Age-related change in executive function: developmental trends and a latent variable analysis. *Neuropsychologia* 44 2017–2036. 10.1016/j.neuropsychologia.2006.01.010 16527316

[B38] Johnston C. (1996). Parent characteristics and parent-child interactions in families of nonproblem children and ADHD children with higher and lower levels of oppositional-defiant behaviour. *J. Abnorm. Child Psychol.* 24 85–104. 10.1007/BF01448375 8833030

[B39] Jurado M. B. Rosselli M. (2007). The elusive nature of executive functions: a review of our current understanding. *Neuropsychol. Rev.* 17 213–233. 10.1007/s11065-007-9040-z 17786559

[B40] Kemper S. Vandeputte S. Rice K. Cheung H. Gubarchuk J. (1995). Speech adjustments to aging during a referential communication task. *J. Lang. Soc. Psychol.* 14 40–59. 10.1177/0261927X95141003

[B41] Kenny D. A. Kashy D. A. Cook W. L. (2006). *Dyadic Data Analysis.* New York, NY: Guilford.

[B42] Kim O. H. Kaiser A. P. (2000). Language characteristics of children with ADHD. *Commun. Disord. Q.* 21 154–165. 10.1177/152574010002100304

[B43] Kitamura C. Burnham D. (2003). Pitch and communicative intent in mother’s speech: adjustments for age and sex in the first year. *Infancy* 4 85–110. 10.1207/S15327078IN0401_5

[B44] Kitamura C. Lam C. (2009). Age-specific preferences for infant-directed affective intent. *Infancy* 14 77–100. 10.1080/15250000802569777 32693467

[B45] Kooij S. J. J. Conners K. C. Goto T. Tanaka Y. Williams D. Allen A. J. (2013). Validity of Conners’ adult attention-deficit/hyperactivity disorder rating scale investigator rated: screening version in patients from within and outside of Europe. *Psychiatry Res.* 208 94–96. 10.1016/j.psychres.2012.12.003 23318025

[B46] Kryski K. R. Mash E. J. Ninowski J. E. (2010). Maternal symptoms of attention-deficit/hyperactivity disorder and maternal language: implications for infant language and development. *J. Child Fam. Stud.* 19 270–277. 10.1007/s10826-009-9294-6

[B47] Lambek R. Tannock R. Dalsgaard S. Trillingsgaard A. Damm D. Thomsen P. (2011). Executive dysfunction in school-age children with ADHD. *J. Atten. Disord.* 15 646–655. 10.1177/1087054710370935 20858784

[B48] Landau S. Milich R. (1988). Social communication patterns of attention-deficit-disordered boys. *J. Abnorm. Child Psychol.* 16 69–81. 10.1007/BF00910501 3361031

[B49] Leonard M. A. Milich R. Lorch E. P. (2011). The role of pragmatic language use in mediating the relation between hyperactivity and inattention and social skills problems. *J. Speech Lang. Hear Res.* 54 567–579. 10.1044/1092-4388(2010/10-0058) 20719870

[B50] Locke K. D. Sadler P. (2007). Self-efficacy, values, and complementarity in dyadic interactions: integrating interpersonal and social-cognitive theory. *Pers. Soc. Psychol. Bull.* 33 94–109. 10.1177/0146167206293375 17178933

[B51] Martinussen R. Hayden J. Hogg-Johnson S. Tannock R. (2005). A meta-analysis of working memory impairments in children with attention-deficit/hyperactivity disorder. *J. Am. Acad. Child Adolesc. Psychiatry* 44 377–384. 10.1097/01.chi.0000153228.72591.73 15782085

[B52] Mash E. J. Johnston C. (1982). A comparison of the mother-child interactions of younger and older hyperactive and normal children. *Child Dev.* 53 1371–1381. 10.2307/1129028 7140436

[B53] Miyake A. Friedman N. P. Emerson M. J. Witzki A. H. Howerter A. (2000). The unity and diversity of executive functions and their contributions to complex “frontal lobe” tasks: a latent variable analysis. *Cogn. Psychol.* 41 49–100. 10.1006/cogp.1999.0734 10945922

[B54] Morton J. B. Trehub S. E. (2001). Children’s understanding of emotion in speech. *Child Dev.* 72 834–843. 10.1111/1467-8624.00318 11405585

[B55] Nigg J. T. (2001). Is ADHD an inhibitory disorder? *Psychol. Bull.* 127 571–598. 10.1037/0033-2909.127.5.571 11548968

[B56] Nilsen E. S. Fecica A. M. (2011). A model of communicative perspective-taking for typical and atypical populations of children. *Dev. Rev.* 31 55–78. 10.1016/j.dr.2011.07.001

[B57] Nilsen E. S. Graham S. A. (2009). The relations between children’s communicative perspective-taking and executive functioning. *Cogn. Psychol.* 58 220–249. 10.1016/j.cogpsych.2008.07.002 18809176

[B58] Nilsen E. S. Graham S. A. (2012). The development of preschoolers’ appreciation of communicative ambiguity. *Child Dev.* 83 1400–1415. 10.1111/j.1467-8624.2012.01762.x 22497242

[B59] Nilsen E. S. Mangal L. MacDonald K. (2013). Referential communication in children with ADHD: the role of a listener. *J. Speech Lang. Hear Res.* 56 590–603. 10.1044/1092-4388(2012/12-0013) 22988288

[B60] Nilsen E. S. Varghese A. Fecica A. Xu Z. (2015). Children’s production of referential statements. An examination of the role of ADHD traits and executive functioning. *J. Cogn. Dev.* 36 68–82. 10.1017/S0305000911000432

[B61] Normand S. Flora D. B. Toplak M. E. Tannock R. (2012). Evidence for a general ADHD factor from a longitudinal general school population study. *J. Abnorm. Child Psychol.* 40 555–567. 10.1007/s10802-011-9584-5 22033884

[B62] Oerlemans A. M. van der Meer J. M. Steijn D. J. de Ruiter S. W. Bruijn Y. G. Sonneville L. M. (2014). Recognition of facial emotion and affective prosody in children with ASD (+ADHD) and their unaffected siblings. *Eur. J. Child Adolesc. Psychiatry* 23 257–271. 10.1007/s00787-013-0446-2 23824472

[B63] O’Neill D. K. (2007). The language use inventory for young children: a parent-report measure of pragmatic language development for 18- to 47-month-old children. *J. Speech Lang. Hear Res.* 50 214–228. 10.1044/1092-4388(2007/017) 17344560

[B64] Pardo J. S. (2013). Measuring phonetic convergence in speech production. *Front. Psychol.* 4:559 10.3389/fpsyg.2013.00559 PMC3753450 23986738

[B65] Psychogiou L. Daley D. Thompson M. Sonuga-Barke E. (2007). Testing the interactive effect of parent and child ADHD on parenting in mothers and fathers: a further test of the similarity-fit hypothesis. *Br. J. Dev. Psychol.* 25 419–433. 10.1348/026151006X170281

[B66] Psychogiou L. Daley D. M. Thompson M. J. Sonuga-Barke E. J. S. (2008). Do maternal attention-deficit/hyperactivity disorder symptoms exacerbate or ameliorate the negative effect of child attention-deficit/hyperactivity disorder symptoms on parenting? *Dev. Psychopathol.* 20 121–137. 10.1017/S0954579408000060 18211731

[B67] Purvis K. L. Tannock R. (1997). Language abilities in children with attention deficit hyperactivity disorder, reading disabilities, and normal controls. *J. Abnorm. Child Psychol.* 25 133–144. 10.1023/A:1025731529006 9109030

[B68] Reitan R. M. Wolfson D. (1985). *The Halstead-Reitan Neuropsychological Test Battery: Theory and Clinical Interpretation.* Tucson, AZ: Neuropsychology Press.

[B69] Roid G. H. (2003). *Stanford-Binet Intelligence Scales, (SB5).* Rolling Meadows, IL: Riverside.

[B70] Sánchez-Cubillo I. Periáñez J. A. Drover-Roig D. Rodríguez-Sánchez J. M. Ríos-Lago M. Tirapu J. (2009). Construct validity of the trail making test: role of task-switching, working memory, inhibition/interference control, and visuomotor abilities. *J. Int. Neuropsychol. Soc.* 15 438–450. 10.1017/S1355617709090626 19402930

[B71] Scouting Web (2013). *Survival: A Simulation Game.* Available at: http://www.epilogsys.com/scoutingweb/SubPages/SurvivalGame.htm

[B72] Sonuga-Barke E. J. S. Dalen L. Daley D. Remington B. (2002). Are planning, working memory, and inhibition associated with individual differences in preschool ADHD traits? *Dev. Neuropsychol.* 21 255–272. 10.1207/S15326942DN2103_3 12233938

[B73] Strauss E. Sherman E. M. S. Spreen O. (2006). *A Compendium of Neuropsychological Tests: Administration, Norms, and Commentary*, 3rd Edn New York, NY: Oxford University Press.

[B74] Stroop J. R. (1935). Studies of interference in serial verbal reactions. *J. Exp. Psychol.* 18 643–662. 10.1037/h0054651

[B75] Struchen M. A. Clark A. N. Sander A. M. Mills M. R. Evans G. Kurtz D. (2008). Relation of executive functioning and social communication measures to functional outcomes following traumatic brain injury. *NeuroRehabilitation* 23 185–198. 18525140

[B76] Swanson J. M. (1992). *School-Based Assessments and Interventions for ADD Students.* Irvine, CA: KC Publishing.

[B77] Thorell L. B. Wåhlstedt C. (2006). Executive functioning deficits in relation to symptoms of ADHD and/or ODD in preschool children. *Infant Child Dev.* 15 503–518. 10.1002/icd.475

[B78] Tomasello M. Mannle S. (1985). Pragmatics of sibling speech to one-year-olds. *Child Dev.* 56 911–917. 10.2307/1130103

[B79] Tracey T. J. G. (2004). Levels of interpersonal complementarity: a simplex representation. *Pers. Soc. Psychol. Bull.* 30 1211–1225. 10.1177/0146167204264075 15359023

[B80] Uekermann J. Abdel-Hamid M. Lehmkämper C. Vollmoeller W. Daum R. (2008). Perception of affective prosody in major depression: a link to executive functions? *J. Int. Neuropsychol. Soc.* 14 552–561. 10.1017/S1355617708080740 18577284

[B81] Uekermann J. Kraemer M. Abdel-Hamid M. Schimmelmann B. G. Hebebrand J. Daum I. (2010). Social cognition in attention-deficit hyperactivity disorder (ADHD). *Neurosci. Biobehav. Rev.* 34 734–743. 10.1016/j.neubiorev.2009.10.009 19857516

[B82] Varghese A. Nilsen E. (2015). Prosody influences children’s judgments of appropriate communication styles. *Poster presented at Society for Research in Child Development (SRCD) Biennial Meeting*, Philadelphia, PA.

[B83] VideoLAN (2012). *VLC Media Player (Version 2.0.0) [computer software].* Available from http://www.videolan.org/vlc/

[B84] Wardlow L. (2013). Individual differences in speakers’ perspective taking: The roles of executive control and working memory. *Psychon. Bull. Rev.* 20 766–772. 10.3758/s13423-013-0396-1 23408369 PMC3860364

[B85] Warren-Leubecker A. Bohannon J. N. (1984). Intonation patterns in child-directed speech: mother-father differences. *Child Dev.* 55 1379–1385. 10.2307/1130007

[B86] Weppelman T. L. Bostow A. Schiffer R. Elbert-Perez E. Newman R. S. (2003). Children’s use of the prosodic characteristics of infant-directed speech. *Lang. Commun.* 23 63–80. 10.1016/S0271-5309(01)00023-4

[B87] Whalen C. K. Henker B. Collins B. E. McAuliffe S. Vaux A. (1979). Peer interaction in a structured communication task: comparisons of normal and hyperactive boys and of methylphenidate (Ritalin) and placebo effects. *Child Dev.* 50 388–401. 10.2307/1129414 487880

[B88] Willcutt E. G. Doyle A. E. Nigg J. T. Faraone S. V. Pennington B. F. (2005). Validity of the executive function theory of attention-deficit/hyperactivity disorder: a meta-analytic review. *Biol. Psychiatry* 57 1336–1346. 10.1016/j.biopsych.2005.02.006 15950006

[B89] Wymbs B. T. Wymbs F. A. Dawson A. E. (2015). Child ADHD and ODD behavior interacts with parent ADHD symptoms to worsen parenting and interparental communication. *J. Abnorm. Child Psychol.* 43 107–119. 10.1007/s10802-014-9887-4 24882503

